# Antibiofilm and antivirulence efficacy of myrtenol enhances the antibiotic susceptibility of *Acinetobacter baumannii*

**DOI:** 10.1038/s41598-020-79128-x

**Published:** 2020-12-15

**Authors:** Anthonymuthu Selvaraj, Alaguvel Valliammai, Chandran Sivasankar, Manokaran Suba, Ganeshkumar Sakthivel, Shunmugiah Karutha Pandian

**Affiliations:** 1 Department of Biotechnology, Alagappa University, Karaikudi, Tamil Nadu 630 003 India; 2 Department of Food Science and Technology, Pondicherry University, Pondicherry, India

**Keywords:** Biological techniques, Biotechnology, Microbiology, Pathogenesis

## Abstract

*Acinetobacter baumannii* (AB) is rising as a human pathogen of critical priority worldwide as it is the leading cause of chronic opportunistic infections in healthcare settings and the condition is ineradicable with antibiotic therapy. AB possesses the ability to form biofilm on abiotic as well as biotic surfaces which plays a major role in its pathogenesis and resistance in clinical settings. Hence, the demand for an alternative therapy to combat the biofilm-associated infections is increasing. The present study explored the antibiofilm potential of myrtenol, a bicyclic monoterpene present in various plants against reference and clinical strains of AB. Myrtenol (200 μg/mL) exhibited a strong antibiofilm activity without exerting any harmful effect on growth and metabolic viability of AB strains. Microscopic analyses confirmed the reduction in the biofilm thickness and surface coverage upon myrtenol treatment. Especially, myrtenol was found to be effective in disrupting the mature biofilms of tested AB strains. Furthermore, myrtenol inhibited the biofilm-associated virulence factors of AB strains such as extracellular polysaccharide, cell surface hydrophobicity, oxidant resistance, swarming and twitching motility. Transcriptional analysis unveiled the suppression of the biofilm-associated genes such as *bfmR*, *csuA/B*, *bap*, *ompA*, *pgaA*, *pgaC,* and *katE* by myrtenol. Notably, myrtenol improved the susceptibility of AB strains towards conventional antibiotics such as amikacin, ciprofloxacin, gentamicin and trimethoprim. Thus, the present study demonstrates the therapeutic potential of myrtenol against biofilm-associated infections of AB.

## Introduction

*Acinetobacter baumannii* (AB) is a Gram-negative, coccobacillus, opportunistic human pathogen and it most frequently causes nosocomial infections such as ventilator-associated pneumonia, bacteraemia, endocarditis, meningitis, wound infections, respiratory tract and urinary tract infections. It has been listed as one of the pathogens of “ESKAPE” bacteria by Infectious Diseases Society of America (IDSA) because of its resistance to conventional antibiotics^1^. According to the World health organization (WHO) report of 2017, it comes under the critical level in the priority list of multidrug-resistant (MDR) bacteria with extreme threat to human health and it increases the need for the development of new antimicrobial treatments^2^. Resistance capacity of AB is increasing enormously against b-lactams, carbapenems, fluoroquinolones and aminoglycosides worldwide^3^. AB is notorious pathogen due to its ability to produce several virulence factors such as phospholipase, hemolytic activity, siderophore production and acinetobactin synthesis. In addition, AB acquires drug resistance by various mechanisms including quorum sensing, biofilm formation, alteration of outer membrane, secretion systems and efflux pump. Quorum sensing in AB is a two component regulatory system comprising AbaI/AbaR which is homologous to the prototype LuxI/LuxR system of Gram-negative bacteria. AbaI is a sensor protein encoded by abaI which synthesizes signalling molecules also known as acyl homoserine lactones (AHL) and AbaR is a receptor protein encoded by abaR which binds to the AHL. Once activated by AHL, AbaR induces downstream signalling pathways which control the expression of various virulence factors (ompA, AdeFGH efflux pump, PNAG and type I pili) and biofilm formation. Among various virulence mechanisms, biofilm formation is foremost reason for antibiotic resistance and pathogenesis of AB^4,5^.

Biofilm is the three dimensional structural community of bacterial cells adhered to each other as well as to substratum by an extracellular matrix containing polysaccharides, proteins and nucleic acids. Biofilm mode of lifestyle increases the resistance to antibiotics and hosts innate immune system, persistent adherence onto biotic surfaces and also the infectious disease-causing ability of bacteria. Furthermore, chronic biofilm causes severe health issues especially in patients with prosthetic medical devices^6^. Furthermore, several virulence factors are associated with AB biofilms such as biofilm-associated protein (Bap), two-component system (BfmS/BfmR), chaperon-usher pilus (csuA/B, A, B, C, D, and E), poly-β-(1,6)-*N*-acetyl glucosamine (PNAG) and outer membrane protein A (OmpA)^7,8^. Therefore, targeting the biofilm formation of AB is the effective way to control antibiotic-resistance and biofilm-associated infections. Currently, the need for a novel therapeutic strategy to combat biofilm-associated infection of AB has gained more attention. Antibiofilm agents attenuate adherence and virulence factors of pathogen instead of affecting its growth and hence the possibility of resistance development is much lower. In addition, antibiofilm therapy enhances the sensitivity of bacteria to antibiotics and the host immune system. This indicates the necessity of antibiofilm therapy and also the importance of the discovery of novel antibiofilm agents^9^. Previously, numerous antibiofilm agents from natural sources have been explored against AB biofilms such as chimeric peptides, curcumin, magainin, honokiol and magnolol^10–13^. Myrtenol (PubChem CID: 10582), a bicyclic Monoterpene, is derived from *Myrtus communis*, *Cyperus rotundus*, *Tanacetum vulgare*, *Dendroctonus armandi* and *Pinus armandii*^14–17^*.* WHO expert committee on food additives recognized myrtenol as a safe flavoring ingredient. It has been well known for its food flavouring potential, fragrance property, non-toxicity and various biological activities such as anti-inflammatory, anti-Alzheimer’s, antioxidant, anticancer, gastroprotective activity, anxiolytic, antimicrobial and antibiofilm activity^18–22^. Thus, the present study for the first time evaluated the effect of myrtenol on biofilm associated virulence factors of AB.

## Results

### Effect of myrtenol on the growth and biofilm formation of AB

**Figure 1:**
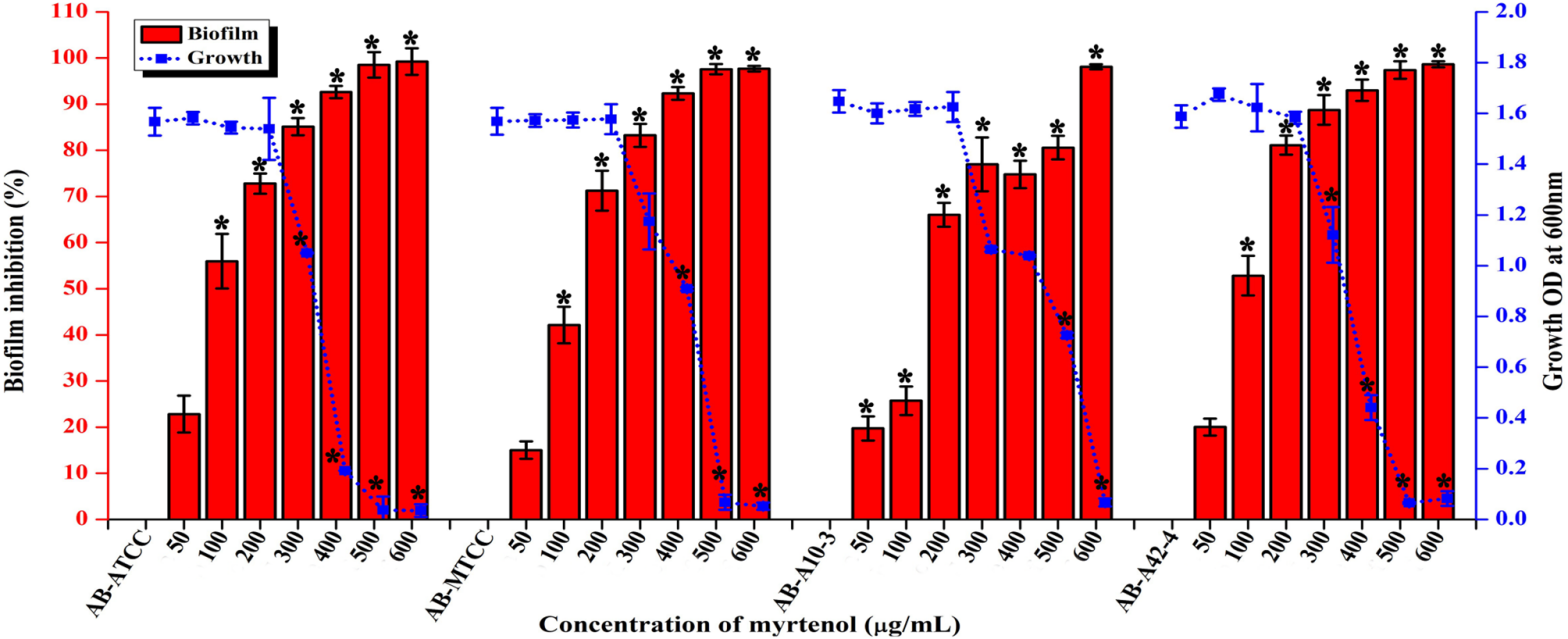
Effect of myrtenol at various concentrations (50–600 μg/mL) on the growth and biofilm formation of AB-ATCC 19606, AB-MTCC 9826, AB-A10-3 and AB-A42-4. Error bars and asterisks indicate the SD and statistical significance (*p* < 0.05), respectively.

To determine the minimum inhibitory concentration (MIC) and minimum biofilm inhibitory concentration (MBIC) of myrtenol against AB strains, microtiter plate method and crystal violet quantification assay were performed, respectively. The result showed that MIC of myrtenol was 500 μg/mL for AB-ATCC 19606, AB-MTCC 9829, AB A10-3 and 600 μg/mL for AB A42-4 strain (Fig. 1). MBIC can be defined as the minimum concentration of drug that exhibits greater than 50% of biofilm inhibition without affecting growth. Myrtenol at a concentration of 200 μg/mL showed strong antibiofilm activity against AB-ATCC 19606 (72%), AB-MTCC 9829 (70%), AB A10-3 (66%) and AB A42-4 (80%) without affecting growth (Fig. 1). Hence, 200 μg/mL was fixed as the MBIC for all tested strains.

### Microscopic visualization of AB biofilm

**Figure 2:**
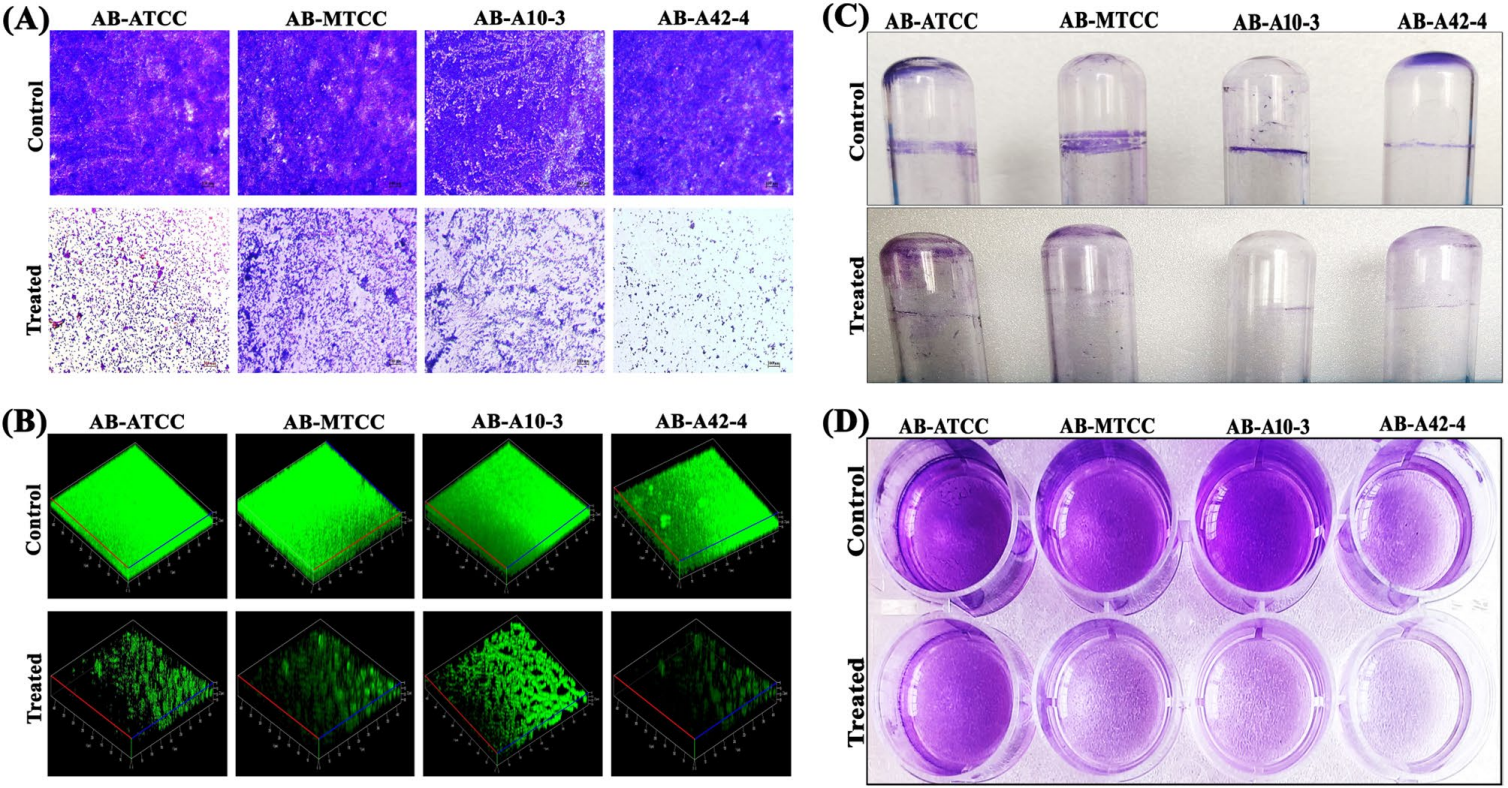
(**A**) Light and (**B**) CLSM images representing the antibiofilm efficacy of myrtenol at 200 μg/mL concentration against AB-ATCC 19606, AB-MTCC 9826, AB-A10-3 and AB-A42-4. Inhibitory potential of myrtenol on (**C**) ring biofilm formation and (**D**) adherence on polystyrene surface by AB-ATCC 19606, AB-MTCC 9826, AB-A10-3 and AB-A42-4.

In light microscopic analysis, highly aggregated and well-structured biofilm formation was observed in AB control samples whereas in myrtenol treated samples significant reduction in biofilm with dispersed cells was observed in all the tested AB strains (Fig. 2A). To assess the effect of myrtenol on the three dimensional biofilm architecture of AB, confocal laser scanning microscopy (CLSM) analysis was performed. Multi-layered biofilm and high biofilm biomass was observed in control samples. In contrast, great reduction in biofilm biomass, surface covered area and thickness was observed in myrtenol (200 μg/mL) treated AB strains (Fig. 2B). Effect of myrtenol on biofilm thickness, biomass and surface to volume ratio was assessed through Comstat analysis. Results revealed that myrtenol treatment notably affected the tested parameters such as biomass, maximum thickness and surface to volume ratio (Table 1).

**Table 1:** Comstat analysis of biofilms formed by AB strains in the absence and presence of myrtenol.

AB strains	Biomass (μm^3^/μm^2^)		Maximum thickness (μm)		Surface to volume ratio (μm^2^/μm^3^)	
	Control	Treated	Control	Treated	Control	Treated
AB-ATCC	18.68 ± 0.85	8.64 ± 1.20	19.90 ± 0.90	4.87 ± 0.53	1.05 ± 0.09	2.15 ± 0.24
AB-MTCC	28.63 ± 0.90	15.43 ± 0.92	55.41 ± 1.07	21.91 ± 1.38	0.76 ± 0.04	1.92 ± 0.15
AB-A10-3	23.18 ± 0.86	9.67 ± 1.16	30.00 ± 1.05	16.80 ± 1.48	0.53 ± 0.23	1.95 ± 0.21
AB-A42-4	24.44 ± 1.54	12.57 ± 0.62	36.56 ± 2.44	19.10 ± 2.50	0.71 ± 0.00	1.39 ± 0.16

### Effect of myrtenol on AB ring biofilm formation and adherence on polystyrene surface

AB has ability to form biofilm at air–liquid interfaces by adhering to the top portion of liquid medium. Therefore, the effect of myrtenol on ring biofilm formation of AB was assessed. Myrtenol reduced the air–liquid interface biofilm formation of all the tested AB strains (Fig. 2C). In addition, myrtenol effectively inhibited the attachment of AB strains on polystyrene surface as exhibited by crystal violet staining (Fig. 2D).

### Myrtenol disrupts mature biofilm formed by AB

**Figure 3:**
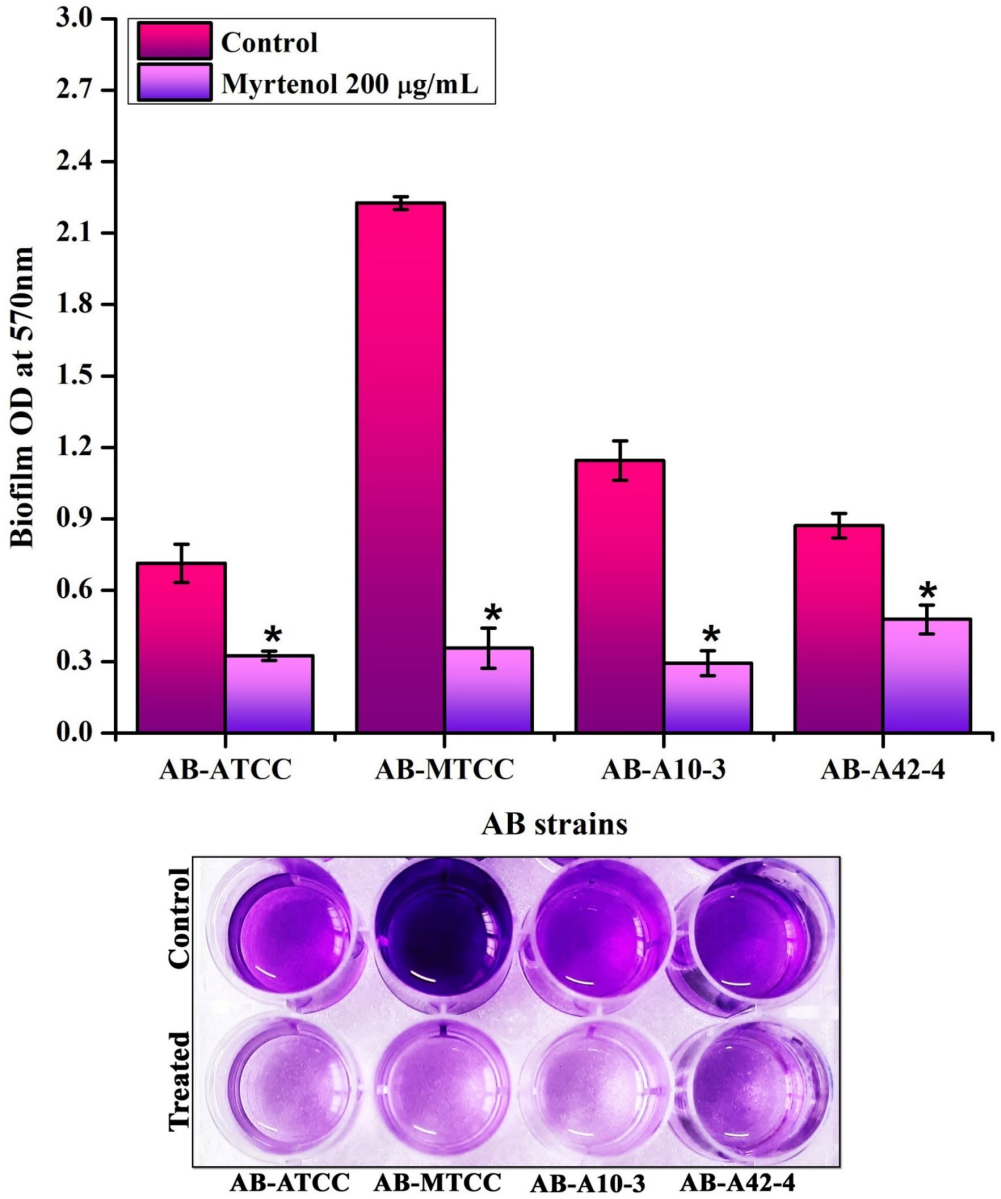
Biofilm disrupting potential of myrtenol (200 μg/mL) on preformed biofilms of AB-ATCC 19606, AB-MTCC 9826, AB-A10-3 and AB-A42-4. Bottom panel depicts the mature biofilm disruptive potential of myrtenol against AB strains. Error bars and asterisks indicate the SD and statistical significance (*p* < 0.05), respectively.

To check the biofilm disrupting ability of myrtenol, mature biofilm disruption assay was performed. Results revealed the potential of myrtenol in disrupting the preformed biofilms of all the tested AB strains (Fig. 3).

### Non-antibacterial effect of myrtenol on AB

**Figure 4:**
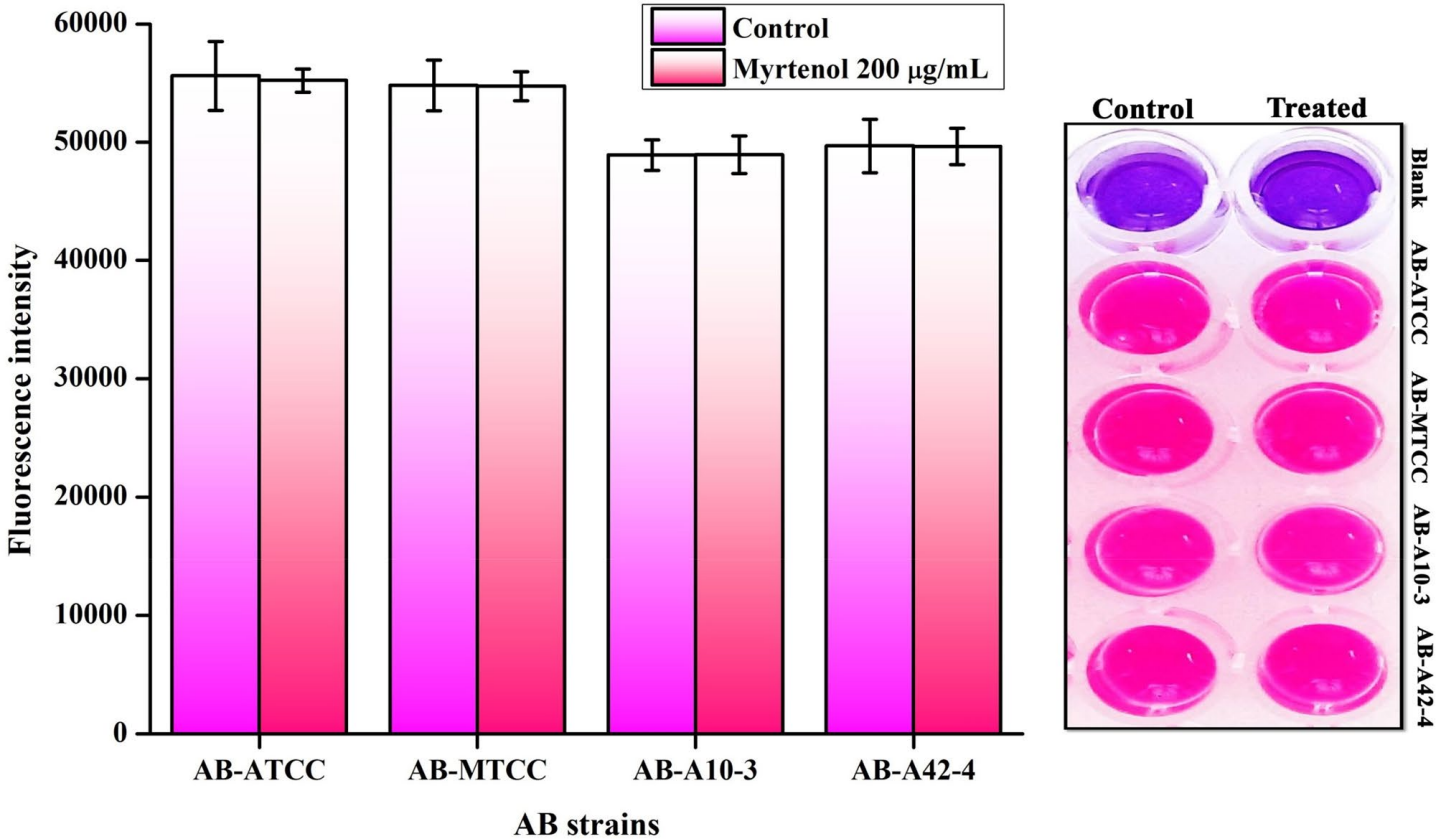
Effect of myrtenol (200 μg/mL) on viability of AB-ATCC 19606, AB-MTCC 9826, AB-A10-3 and AB-A42-4 as assessed by Alamar blue reduction assay. Error bars indicate the SD.

To determine the non-antibacterial effect of myrtenol against both biofilm and planktonic cells of AB strains, Alamar blue assay was performed. Alamar blue assay revealed that myrtenol does not affect the cell viability of tested AB strains as results showed no significant variance in the fluorescence intensity of control and myrtenol treated sample (Fig. 4).

### Myrtenol affects extracellular polysaccharides (EPS) production and cell surface hydrophobicity (CSH) of AB

**Figure 5:**
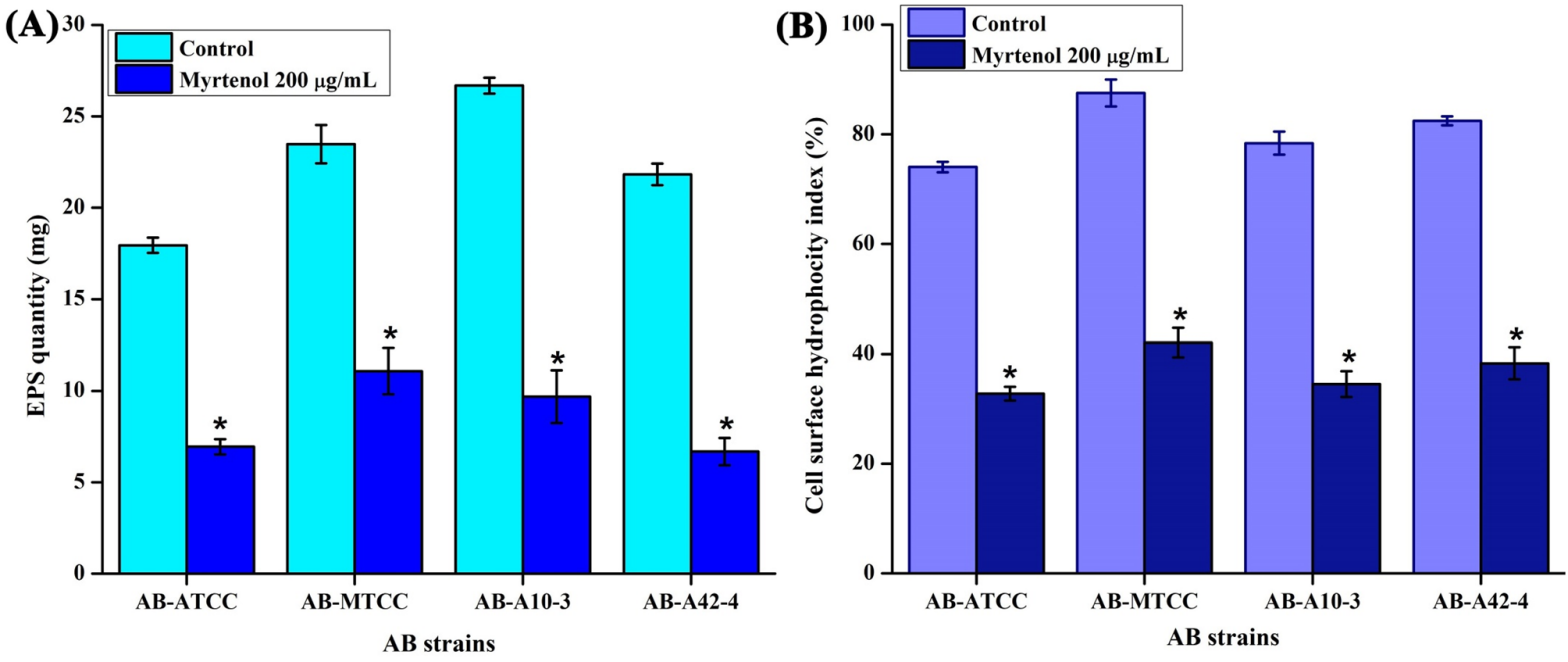
Inhibitory effect of myrtenol (200 μg/mL) on (**A**) EPS production and (**B**) CSH of AB-ATCC 19606, AB-MTCC 9826, AB-A10-3 and AB-A42-4. Error bars and asterisks indicate the SD and statistical significance (*p* < 0.05), respectively.

The EPS production in AB is directly associated with biofilm formation and maintains the biofilm integrity. The quantitative mass analysis of EPS production in myrtenol treated samples indicated a significant reduction of EPS. At MBIC (200 μg/mL) of myrtenol, more than 60% reduction in EPS was observed in all the tested AB strains when compared to control sample (Fig. 5A). Furthermore, CSH of AB is playing significant role in its adherence to both biotic and abiotic surfaces. Hence, the effect of myrtenol on CSH of AB was examined and result showed a constant reduction in CSH of myrtenol treated AB strains when compared to the control sample (Fig. 5B). This result confirmed that myrtenol affects the CSH of AB.

### Myrtenol impedes the motility of AB

**Figure 6:**
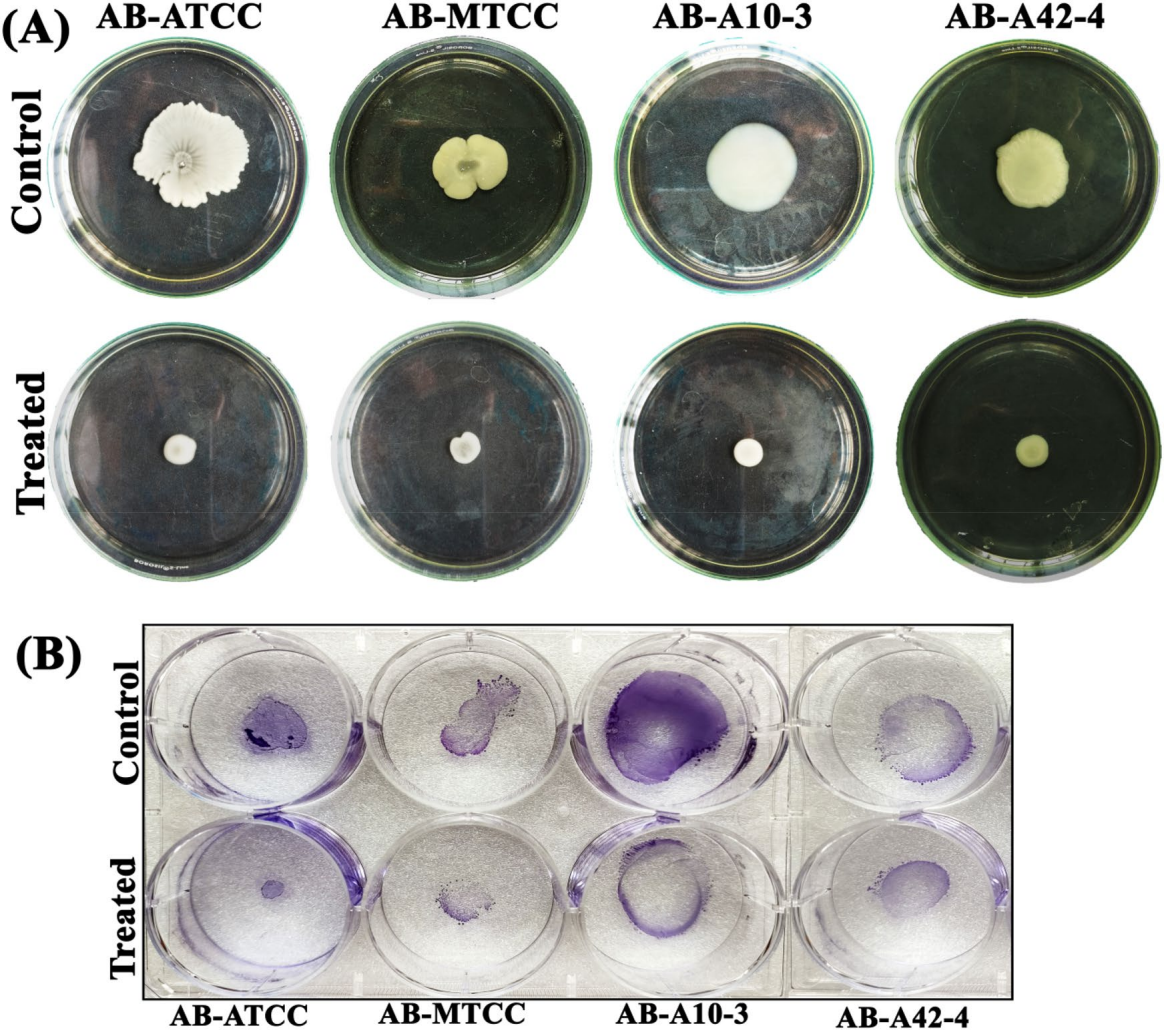
Effect of myrtenol (200 μg/mL) on (**A**) swarming and (**B**) twitching motility of AB-ATCC 19606, AB-MTCC 9826, AB-A10-3 and AB-A42-4.

AB is exploiting the flagellum and pili for motility, environmental survival and colonization on different surfaces. Therefore, swarming and twitching motility assays were performed in the absence and presence of myrtenol. Interestingly, myrtenol inhibited the swarming and twitching motility of AB to a great extent. At 200 μg/mL concentration of myrtenol, maximum reduction in swarming motility of AB strains was observed compared to high swarming motility of control sample (Fig. 6A). Twitching motility was also found to be inhibited at 200 μg/mL concentration of myrtenol when compared to the control samples (Fig. 6B).

### Myrtenol reduces the resistance of AB to oxidants

**Figure 7:**
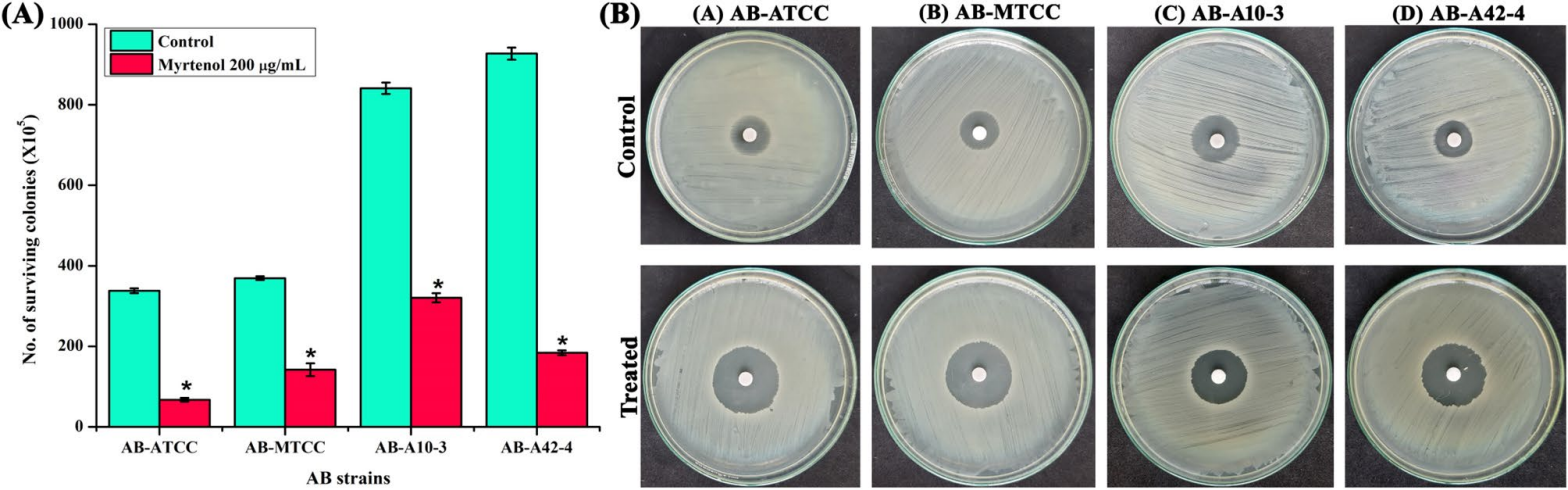
(**A**) Effect of myrtenol (200 μg/mL) on the survival of AB-ATCC 19606, AB-MTCC 9826, AB-A10-3 and AB-A42-4 in the presence of H_2_O_2_ and (**B**) plate images showing the effect of myrtenol on the sensitivity of AB strains to H_2_O_2_ by disc diffusion assays. Error bars and asterisks indicate the SD and statistical significance (*p* < 0.05), respectively.

Reactive oxygen species (ROS) generated by host immune cells are responsible for innate immune clearance of bacterial pathogen. Therefore, hydrogen peroxide (H_2_O_2_) sensitivity assay was performed to assess the effect of myrtenol on sensitivity of AB to ROS. From the result it was observed that myrtenol at 200 μg/mL significantly reduced the survival of AB strains when compared to untreated control sample (Fig. 7A). The result of H_2_O_2_ disc diffusion assay also showed the increased zone of clearance in the presence of myrtenol (200 μg/mL) when compared to zone of clearance of AB control cells. The increasing zone of clearance observed in myrtenol treated samples indicates the enhanced sensitivity of AB to ROS (Fig. 7B).

### Myrtenol modulates the expression of biofilm associated genes in AB

**Figure 8:**
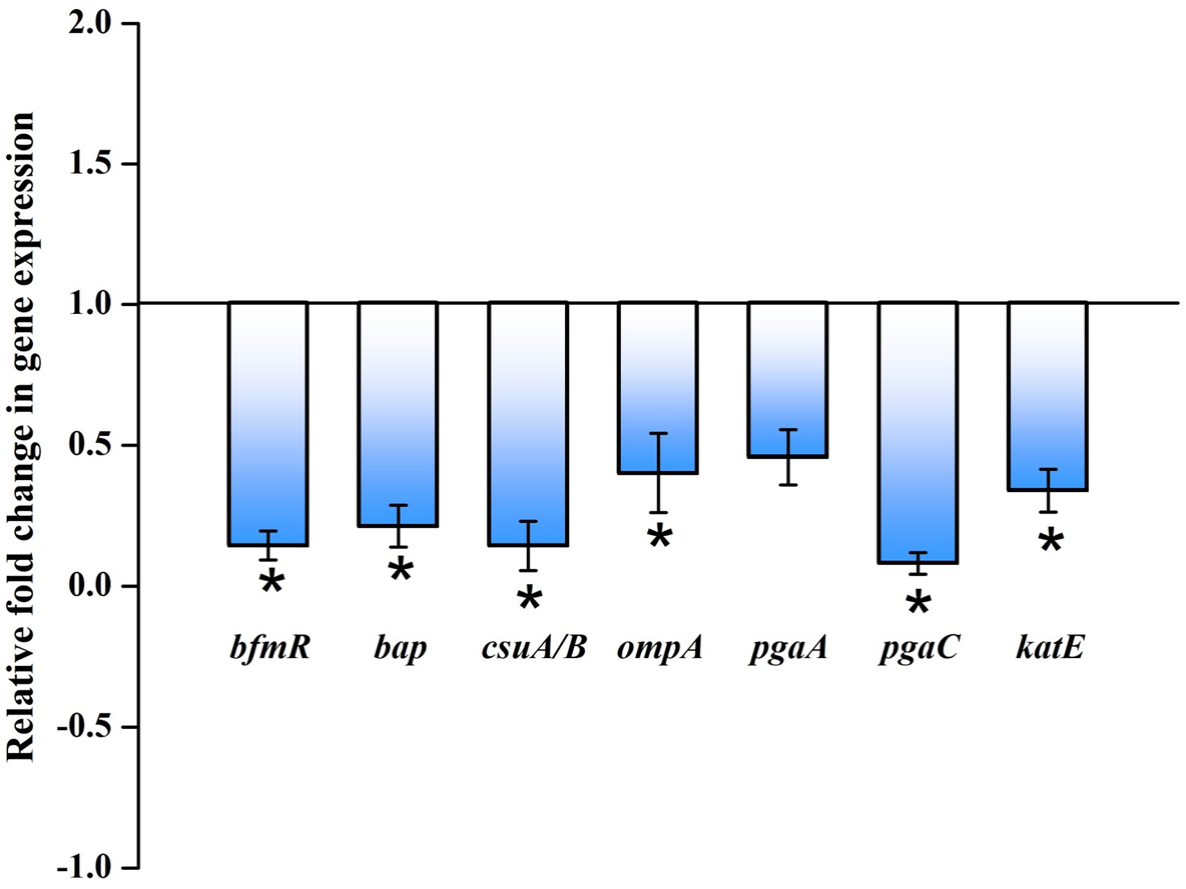
Effect of myrtenol (200 μg/mL) on expression profile of genes involved in biofilm formation in AB-ATCC 19606. Error bars and asterisks indicate the SD and statistical significance (*p* < 0.05), respectively.

qPCR analysis was performed to assess the effect of myrtenol on expression of biofilm associated genes in AB. The result revealed a significant reduction in the expression of *bfmR*, *bap*, *csuA/B*, *ompA*, *pgaA*, *pgaC* and *katE* (Fig. 8).

### Myrtenol improves the antibiotic susceptibility of AB

**Figure 9:**
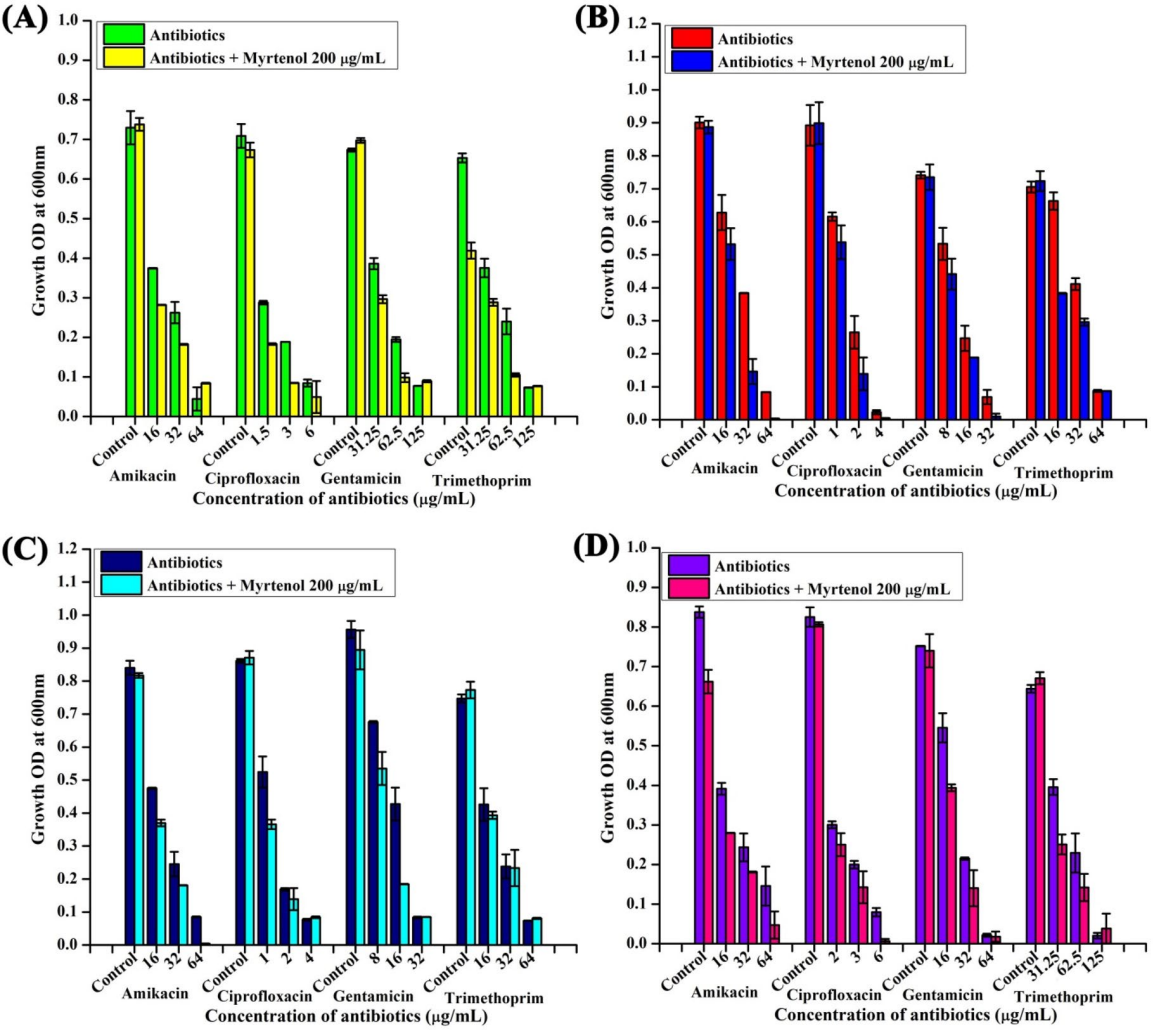
Effect of myrtenol (200 μg/mL) on the susceptibility of AB-ATCC 19606, AB-MTCC 9826, AB-A10-3 and AB-A42-4 to antibiotics. Error bars and asterisks indicate the SD and statistical significance (*p* < 0.05), respectively.

The antibiotics susceptibility of AB was assessed in the absence and presence of myrtenol (200 μg/mL) for which initially MIC of antibiotics such as amikacin, ciprofloxacin, gentamicin and trimethoprim was determined by microbroth dilution method (Supplementary Table 1). Interestingly, addition of myrtenol considerably increased the sensitivity of antibiotics against AB strains (Fig. 9). Further, zone of inhibition of antibiotics was also found to be increased in the presence of myrtenol (Table 2; Supplementary Fig. 1). These results clearly indicate that myrtenol increases the susceptibility of AB strains towards the antibiotics.

**Table 2:** Antibiotic sensitivity pattern of AB strains in the absence and the presence of myrtenol.

	AB-ATCC		AB-MTCC		AB-A10-3		AB-A42-4	
	Control	Treated	Control	Treated	Control	Treated	Control	Treated
Amikacin	9 ± 0	11.83 ± 0.29	13.50 ± 0.50	15.33 ± 0.58	14.67 ± 0.58	17.17 ± 0.29	11.33 ± 0.58	14.0 ± 0
Ciprofloxacin	15.83 ± 0.29	16.67 ± 0.58	17.0 ± 0	18.83 ± 0.29	17.67 ± 0.58	18.83 ± 0.76	13.83 ± 0.29	18.17 ± 0.29
Gentamycin	13 ± 0.29	14 ± 0	14.50 ± 0.50	18.50 ± 0.87	14.67 ± 0.58	16.83 ± 0.76	10.33 ± 0.58	12.33 ± 0.58
Trimethoprim	20.0 ± 0	21.33 ± 0.29	14.83 ± 0.29	20.50 ± 0.50	21.33 ± 0.58	22.17 ± 0.29	17.33 ± 0.58	18.0 ± 0

## Discussion

**Figure 10:**
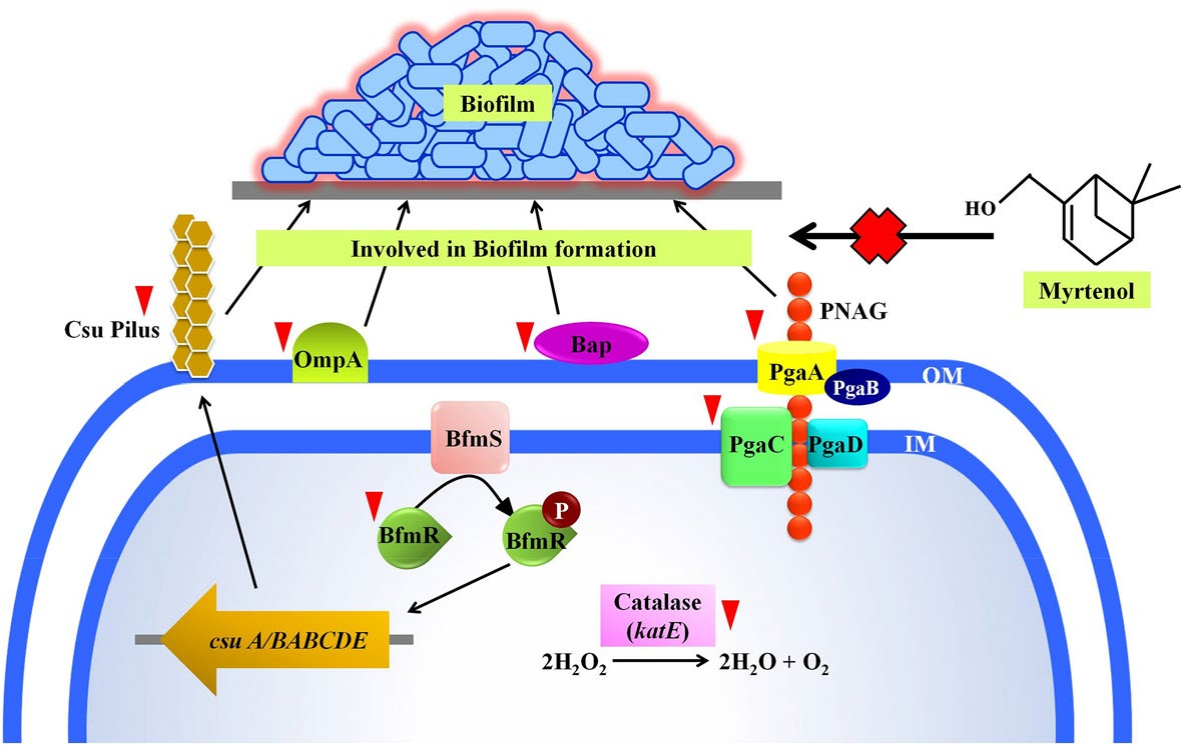
A schematic diagram representing the effect of myrtenol on biofilm associated genes expression in AB. Red coloured inverted triangles indicate down regulation of genes by myrtenol.

The biofilm formation enhances the infectious disease-causing ability of AB in healthcare settings and is also responsible for the emergence of antibiotic resistance. Thus, antibiofilm based approach to fight infectious diseases has gained more attention among the scientific community. Myrtenol is a non-toxic natural molecule and though well known for various biological activities^18–21^, antibiofilm efficacy of myrtenol against AB remains unexplored. In the present study, antibiofilm potential of myrtenol was assessed against two reference strains (AB-ATCC 19606 and AB-MTCC 9829) and two clinical isolates (AB A10-3 and AB A42-4). Crystal violet quantification assay revealed that myrtenol at 200 μg/mL concentration could significantly inhibit biofilm formation of AB-ATCC 19606, AB-MTCC 9829, AB A10-3 and AB A42-4 strains by 72%, 70%, 65% and 80%, respectively. In order to examine the effect of myrtenol on biofilm architecture, light microscopic, CLSM and Comstat analyses were performed and results depicted the substantial reduction in the biofilm covered surface area as well as the thickness of biofilm architecture in myrtenol treated samples compared to the control, where multi-layered biofilm was observed in high quantity. In addition, myrtenol was able to inhibit biofilm formation of AB strains on glass and polystyrene surfaces. Most notably, myrtenol has the potential to disrupt preformed biofilms of AB strains which is very appreciable nature of myrtenol as mature biofilms of AB formed in clinical context is very challenging to eradicate and is responsible for persistence of infection^21^. An ideal antibiofilm agent is expected to have non-fatal effect on bacterial growth and hence the chance of causing any selective pressure on the bacteria is much lower. Consequently, the results of Alamar blue assay unveiled the non-bactericidal effect of myrtenol on AB and non-bactericidal antibiofilm efficacy of myrtenol is highly beneficial in therapeutic aspect. The bacterial biofilm is made up with EPS which acts as barrier to antibiotics and host immune system. Several studies have revealed the important role of EPS in biofilm formation such as protection from environmental stress, providing stability to biofilm matrices and enhanced cell to cell adherence. Thus, inhibiting the production of EPS could increase the sensitivity of bacteria to antibiotics and host innate immune system and eventually it helps to recover biofilm-associated infections^23–25^. Hence, the EPS was extracted from control and myrtenol treated cultures and the results indicated the EPS inhibitory effect of myrtenol. The EPS production influences the CSH of bacteria thereby it enhances bacterial colonization and attachment to biotic and abiotic surfaces. Prior research has shown that the distribution of EPS affects the CSH wherein the quantity of EPS production was directly linked with CSH of cells^26^. Thus, MATH assay was done to assess the effect of EPS reduction on CSH of control and myrtenol treated cells. The result of MATH assay showed significant inhibition in the CSH of myrtenol treated sample which can be corroborated with the above theory and as well as another report^27^. The presence of flagella, pili and fimbriae in AB promotes the bacterial motility and biofilm formation. Flagella and pili are involved in swarming motility and twitching motility, respectively. The fimbriae of AB also helps bacterial motility thereby it enhances the biofilm formation between air–liquid interfaces. AB motility mechanisms employ the initial attachment of pathogen to host cells and support internal colonization during pathogenesis. This kind of adherence potential of AB enhances its survival against conventional antimicrobial agents and the human host immune system^28–30^. Previous study showed that the mutation in a gene (*pilT*) responsible for motility affects biofilm formation and bacterial adherence^31^. Therefore, we assessed the influence of myrtenol on the motility of AB and results of motility assays revealed that myrtenol could profoundly affect both the flagella mediated swarming motility and pili associated twitching motility. AB also forms biofilm on the air–liquid interface and it is also associated with the motility and adherence of AB^32^. As motility of AB was found to be affected in the presence of myrtenol, the air–liquid interface biofilm formation of AB was also expected to get affected. In order to validate this, ring biofilm formation assay was performed and as expected, concentration dependent inhibitory activity of myrtenol on air–liquid interface biofilm formation of AB was observed. In addition, AB has the ability to synthesize antioxidant enzymes such as catalase and superoxide dismutase to neutralize its own reactive oxygen species (ROS) produced during metabolism and ROS generated in the host during phagocytosis. Antioxidant enzyme production in AB is connected with the quorum sensing mediated biofilm formation^33^. Thus, the sensitivity of AB to H_2_O_2_ was analyzed and the obtained results showed that myrtenol treated cells were more susceptible to H_2_O_2_ compared to the control cells. In order to validate the results of phenotypic assays and to identify the mode of antibiofilm activity of myrtenol, qPCR analysis was performed for biofilm-associated genes of AB such as *bfmR*, *bap*, *csuA/B*, *ompA*, *pgaA*, *pgaC* and *katE*^34,35^. In AB, BfmR is a two-component signal transduction system and it also acts as the master regulator of biofilm initiation. In addition, BfmR regulates the expression of the chaperone-usher system (*csuA/BABCDE*) which promotes pili assembly and fimbriae based bacterial motility^36,37^. The qPCR data showed down-regulation of *bfmR* and *csuA* and it was in total agreement with the result of motility and ring biofilm formation assays. Bap of AB is one of the cell surface-associated proteins and involved in biofilm maintenance and maturation. Mutation in the *bap* gene showed the inhibition in the biofilm formation by AB^38,39^. OmpA of AB is associated with several biological processes such as bacterial adherence to different surfaces and antimicrobial resistance. Previous study demonstrated the key role of OmpA on bacterial adherence to human keratinocyte and bronchial epithelial cells^40^. In this study, the results of qPCR analysis showed the down-regulation of *ompA* and *bap* genes which can be connected with results of biofilm quantification and MATH assays. *pgaABCD* locus of AB produces the proteins required for the synthesis of PNAG which is a major polysaccharide component of the biofilm matrix. According to a previous report, the deletion of the pga locus strongly affects the biofilm formation in AB^41^. Thus, the effect of myrtenol on expression of *pgaA* and *pgaC* genes was evaluated through qPCR analysis and result showed down-regulation of *pgaA* and *pgaC* genes and it is in line with the results of EPS quantification assay. The result obtained from the H_2_O_2_ sensitivity assay showed that the resistance of AB to oxidants has been reduced upon myrtenol treatment. Hence, the effect of myrtenol on the expression of the *katE* gene which is responsible for catalase production was tested and it comes under the QS mediated biofilm formation system of AB. The down-regulation of the *katE* gene by myrtenol could be attributed to the improved susceptibility of AB to H_2_O_2_ in the presence of myrtenol^42,43^. On the whole, myrtenol effectively inhibited the biofilm and virulence of AB and the schematic representation of myrtenol targeted virulence factors of AB is depicted in Fig. 10. Combinatorial drug therapy is an emerging strategy to prevent bacterial infections. It has an advantage of decreasing the drug dosage level thereby it reduces drug toxicity and development of antibacterial resistance. Previous studies reported that antibiofilm agents increased the sensitivity of chronic infection-causing bacteria towards antibiotics^44,45^. Hence, the influence of myrtenol treatment on AB sensitivity to antibiotics was evaluated and interestingly myrtenol treatment potentiated the susceptibility of AB to conventional antibiotics namely amikacin, ciprofloxacin, gentamicin and trimethoprim. Ability of myrtenol to enhance the antibacterial activity of commonly used antibiotics is highly appreciable in the clinical context.

## Conclusion

Altogether, the present study for the first time revealed the antibiofilm efficacy of myrtenol against AB reference strains and clinical isolates without affecting the growth. Thus the possibility of drug resistance against myrtenol will be much lower. Apart from inhibiting the biofilm of AB, myrtenol also disrupted the preformed biofilms of AB strains. In addition, myrtenol affected the biofilm-associated adherence factors such as EPS, CSH, swarming and twitching motility as revealed by in vitro assays. Further, qPCR analysis exhibited that myrtenol suppressed the expression of key genes involved in biofilm formation. Especially, the antibiofilm potential of myrtenol enhanced the susceptibility of AB to oxidants and conventional antibiotics. Considering antibiofilm and antibiotic combination effect of myrtenol on AB, it could be a promising therapeutic agent against virulence factors of AB.

## Methods

### Bacterial strains and culture conditions

The bacterial strains used in this study are as follows: AB reference strain ATCC-19606, AB reference strain MTCC-9826, AB clinical isolate A10-3 (GenBank ID: KM099445) and AB clinical isolate A42-4 (GenBank ID: KM099485). Bacterial strains were cultured in tryptic soy broth (TSB) supplemented with 1% sucrose and 0.5% yeast extract (TSBSY) to promote biofilm formation and incubated at 37 °C for 24 h in an orbital shaker (160 rpm).

### Phytochemical

Myrtenol was purchased from Sigma-Aldrich, India and prepared as a 10 mg/mL in methanol for all the experiments. Methanol was included as negative control in all the assays carried out.

### Determination of MIC

MIC of myrtenol against AB was determined using microtiter plate method. A 96-well plate was filled with 200 μL of TSBSY containing one percent of overnight culture of AB (10^8^ cells) and increasing concentrations of myrtenol (50, 100, 200, 400, 500 and 600  μ g/mL) and incubated at 37 °C for 24 h. After incubation, the cell density of the culture was measured at 600 nm using Spectramax M3, Molecular devices, United States^46^.

### Determination of MBIC

One percent overnight culture of AB was used to inoculate 1 mL of TSBSY in 24-well plate without and with myrtenol and incubated at 37 °C for 24 h. After incubation, the cell density was measured at 600 nm. After that, planktonic cells were discarded and the well plate containing biofilm cells was washed using sterile phosphate buffered saline (PBS) to remove unbound cells followed by air drying. The plate was then stained with 0.4% of crystal violet solution for 10 min and washed with PBS to remove excess stain. The plate was destained by 30% glacial acetic acid solution and observed at 570 nm. Finally, the percentage of biofilm inhibition was calculated using following formula: (%) = [(Control OD_570nm_ − Treated OD_570nm_)/Control OD_570nm_] × 100^47^.

### Air–liquid interface biofilm formation assay

The influence of myrtenol on biofilm formation of AB strains at air–liquid interface was assessed by previously described method. Briefly, overnight culture was added to 2 mL of TSBSY medium without and with myrtenol at increasing concentrations (200 μg/mL) in the glass test tubes and incubated for 48 h at 37 °C. After incubation, test tubes were washed thrice with PBS and stained with 0.4% crystal violet solution^48^.

### Microscopic analyses

Biofilm assay was performed on 24-well plate containing glass slides in the absence and presence of myrtenol for 24 h at 37 °C. After that, the glass slides were washed by sterile PBS and allowed to air dry. For light microscopic analysis, the slides were stained with 0.4% crystal violet solution and observed at the magnification of 400× under Nikon Eclipse Ti-S, Tokyo, Japan. For CLSM analysis, acridine orange solution (0.1%) was used to stain the glass slides and observed at the magnification of 200× under Zeiss LSM-710, Carl Zeiss, Oberkochen, Germany. Comstat analysis was performed using Comstat 2 software to analyse the biofilm thickness, biomass and surface to volume ratio of biofilm of AB strains grown in the absence and presence of myrtenol^49,50^.

### Biofilm disruption assay

To assess biofilm disruption by myrtenol, preformed AB biofilms were treated with myrtenol. Briefly, 1% of AB strains were added to wells of MTP containing 1 mL of TSBS and incubated for 24 h at 37 °C. After incubation, medium containing planktonic cells was removed and replaced with fresh TSBS with myrtenol (200 μg/mL) and kept at 37 °C. After 24 h, TSBS was removed and biofilm cells were washed with sterile PBS. Percentage of biofilm disruption was calculated after crystal violet staining as mentioned^21^.

### Cell viability assay

Alamar blue assay was performed to assess the influence of myrtenol on viability of AB strains. Briefly, AB strains were grown in 24 well MTP containing 1 mL of TSBSY medium without and with myrtenol (200 μg/mL) for 24 h at 37 °C. Then, biofilm formed on polystyrene surface was scraped off and included with planktonic cells for centrifugation at 8000 rpm for 10 min. Then, cell pellets were harvested, washed thrice and resuspended in 1 mL of PBS. To the cell suspension (900 μL), 100 μL of Alamar blue substrate (6.5 mg/mL in PBS) was added and incubated at 37 °C for 4 h in the dark condition. Cell viability is directly proportional to reduction of Alamar blue into pink-coloured solution and fluorescence intensity of solution was measured at wavelengths of 590 nm for emission and 560 nm for excitation^21^.

### EPS quantification assay

For EPS quantification, AB cultures (100 mL) were grown in the absence and presence of myrtenol (200 μg/mL) for 24 h at 37 °C. To extract both cell bound and secreted EPS, AB cultures were centrifuged at 10,000 rpm for 15 min to collect cell pellets and cell free culture supernatant (CFCS). The cell pellets from control and myrtenol treated samples were suspended in equal volume of isotonic buffer which contains 10 mM Tris/HCl pH 8.0, 10 mM EDTA, 2.5% NaCl. Samples were incubated at 4 °C for 16 h and centrifuged at 8000 rpm for 10 min to collect cell bound EPS in supernatant which was mixed with already collected CFCS. Then, one volume of CFCS containing cell bound and secreted EPS was added with three volume of ice cold ethanol and kept at − 20 °C overnight. After incubation, the suspension was centrifuged at 12,000 rpm at 4 °C for 15 min to collect precipitated carbohydrates as pellet. Then, the pellet was suspended in 70% ethanol and dried under vacuum. At last, the dried EPS was collected as powder and weighed. Percentage of EPS inhibition was calculated by the following formula^51^: EPS = Control weight − Treated weight /Control weight] × 100.

### Microbial adhesion to hydrocarbons (MATH) assay

The influence of myrtenol on CSH of AB was analyzed by MATH assay^51^. Briefly, AB strains were grown in the absence and presence of myrtenol (50, 100 and 200 μg/mL) and cells were collected, washed with PBS and resuspended in the same. The cell suspensions were adjusted to OD of 0.9 at 600 nm to which 1 mL of toluene was added, vortexed for 2 min and kept at 4 °C for 24 h to allow the separation of two phases. After the separation, the OD of the aqueous phase was measured at 600 nm and CSH was calculated by the following formula: CSH (%) = [1 − (after vortex OD_600nm_/before vortex OD_600nm_)] × 100.

### Motility assay

Swarming and twitching motility assays were performed to evaluate the effect of myrtenol on the motility of AB strains. For swarming motility assay, 0.5% of TSA plates were prepared without and with myrtenol at increasing concentrations (50, 100 and 200 μg/mL) and 5 μL of overnight culture was introduced into the middle region of agar plates and incubated for 72 h at 37 °C. After incubation, motility zone was observed and measured. For twitching motility, overnight culture was stabbed on centre to till bottom of 1% TSA plate using sterile toothpick and incubated at 37 °C for 72 h. Then, agar was discarded carefully and plates were washed by PBS and stained with 0.4% crystal violet solution^52^.

### H_2_O_2_ sensitivity assay

The influence of myrtenol treatment on the sensitivity of AB strains to H_2_O_2_ was assessed. Briefly, AB was allowed to grow in the absence and presence of myrtenol (200 μg/mL) for 24 h at 37 °C and cells were harvested by centrifuging 8000 rpm for 10 min. Then, cells were resuspended in PBS containing 10 mM H_2_O_2_ and incubated for 1 h and surviving colonies were enumerated by serial dilution method. Also, the disc diffusion assay was performed by preparing Muller-Hinton agar (MHA) plates supplemented without and with myrtenol (200 μg/mL) and AB culture was swabbed on the surface of MHA plates. Then, sterile discs placed on the centre of plates and loaded with 10 μL of H_2_O_2_ solution and incubated at 37 °C for 24 h. The diameter of the zone of clearance was observed and measured^48^.

### Quantitative real-time PCR analysis

Total RNA from control and myrtenol (200 μg/mL) treated AB cultures was isolated by Trizol method and reverse transcribed into cDNA using High-capacity cDNA Reverse Transcription kit (Applied Biosystems, USA) as per the manufacturer’s instructions. qPCR analysis was performed on real time PCR (7500 Sequence Detection System, Applied Biosystems Inc. Foster, CA, USA) for biofilm associated virulence genes such as *bfmR*, *bap*, *csuA/B*, *ompA*, *pgaA*, *pgaC*, *katE* and *rplB* using PCR mix (SYBR Green kit, Applied Biosystems, United States) at a predefined ratio. The primers of candidate genes are listed in Table 3. Expression of tested genes was normalized with housekeeping gene (*rplB*) and fold change in gene expression was calculated using 2^(−ΔΔCt)^ method^53^.

**Table 3:** List of primers used for qPCR analysis.

Genes	Forward primer	Reverse primer
*bfmR*	5′-CTGGTAGGTAATGCAGTTCG-3′	5′-GAGAGACCCAAACCATAACC-3′
*bap*	5′-GTACTCCAGCAACGGTTGTA-3′	5′-GAAGGATCTGCTGTATTCCA-3
*csuA/B*	5′-ATGCGGTAAATACTCAAGCA-3′	5′-TCACAGAAATATTGCCACCT-3′
*ompA*	5′-CTCTTGCTGGCTTAAACGTA-3′	5′-GCAATTTCTGGCTTGTATTG-3′
*pgaA*	5′-CACATGGCAAAAAGATGAAT-3′	5′-CGTAGAAACCTCGAACAGTG-3′
*pgaC*	5′-CAGTGGTATGGCGTGATATT-3′	5′-GGTACTGCAACAACACTGGT-3′
*katE*	5′-GTGTCCGGTTCAGGTTTTAC-3′	5′-GGATTCTTGACAGACCCAAC-3′
*rplB*	5′-GGTCGTAATAACAACGGTCA-3′	5′-AATAATGCAATATGCGCTGT-3′

### Antibiotic sensitivity assay

To determine the effect of myrtenol in enhancing the susceptibility of AB to antibiotics, MIC of antibiotics (amikacin, ciprofloxacin, gentamycin and trimethoprim, HiMedia, India) was determined by performing microbroth dilution assay.

Further, combinatorial efficacy of myrtenol with the antibiotics was assessed by growing AB strains in the absence and presence of antibiotics at 1×, 0.5× and 0.25× of MIC. Briefly, 200 μL of TSBSY broth was added to each wells of 96 well plate and 1% overnight culture of AB strains was added and treated without and with 200 μg/mL of myrtenol and various concentrations of antibiotics. Plates were further incubated at 37 °C for 24 h and read at 600 nm.

The disc diffusion assay was also performed as per the Clinical and Laboratory Standards Institute guidelines to determine the antibiotic sensitivity of AB in the presence of myrtenol^54^. Briefly, TSA plates were prepared without and with myrtenol (200 μg/mL). Overnight culture of AB strains were swabbed on the TSA plates and sterile discs were placed on the centre of plates and loaded with MIC of antibiotics such as amikacin, ciprofloxacin, gentamycin and trimethoprim were. Then, plates were incubated at 37 °C for 24 h and the zone of inhibition was measured.

### Statistical analysis

All the experiments were done in three biological replicates with three technical replicates. Data were expressed as mean ± standard deviation (SD) and statistical significance was analyzed by one-way ANOVA method and Duncan’s post hoc test using SPSS 17.0 software package (SPSS Inc., Chicago, IL). *p* < 0.05 was fixed as significant value.

## Supplementary Information

Supplementary Information.
