# Supplementary material for: Antibiofilm and antivirulence efficacy of myrtenol enhances the antibiotic susceptibility of Acinetobacter baumannii
Source: Sci Rep. 2020 Dec 15;10:21975. doi: 10.1038/s41598-020-79128-x (PMC7738676; doi:10.1038/s41598-020-79128-x)
Supplement: Supplementary file 1 — Supplementary Information. [file 41598_2020_79128_MOESM1_ESM.pdf]

Antibiofilm and antivirulence efficacy of myrtenol enhances the antibiotic susceptibility of *Acinetobacter baumannii*

Anthonymuthu Selvaraj[1], Alaguvel Valliammai[1], Ganesh kumar Sakthivel[1], Manokaran Suba[1], Chandran Sivasankar[1,2] and Shunmugiah Karutha Pandian[1*]

[1]Department of Biotechnology, Alagappa University, Karaikudi, Tamil Nadu, India.

[2]Department of Food Science and Technology, Pondicherry University, Pondicherry, India.

***Corresponding Author**

Dr. Shunmugiah Karutha Pandian

Senior Professor and Head

Department of Biotechnology, Alagappa University, Karaikudi - 630 003, Tamil Nadu, India.

Mail ID: pandiansk@gmail.com

Fax: +91 4565 225202

Tel: +91 4565 225215

Supplementary Table 1: MIC of antibiotics against AB strains

Antibiotics	MIC (µg/mL)			
	**AB-ATCC**	**AB-MTCC**	**AB-A10-3**	**AB-A42-4**
**Amikacin**	64	64	64	64
**Ciprofloxacin**	6	4	4	6
**Gentamicin**	125	32	32	64
**Trimethoprim**	125	64	64	125

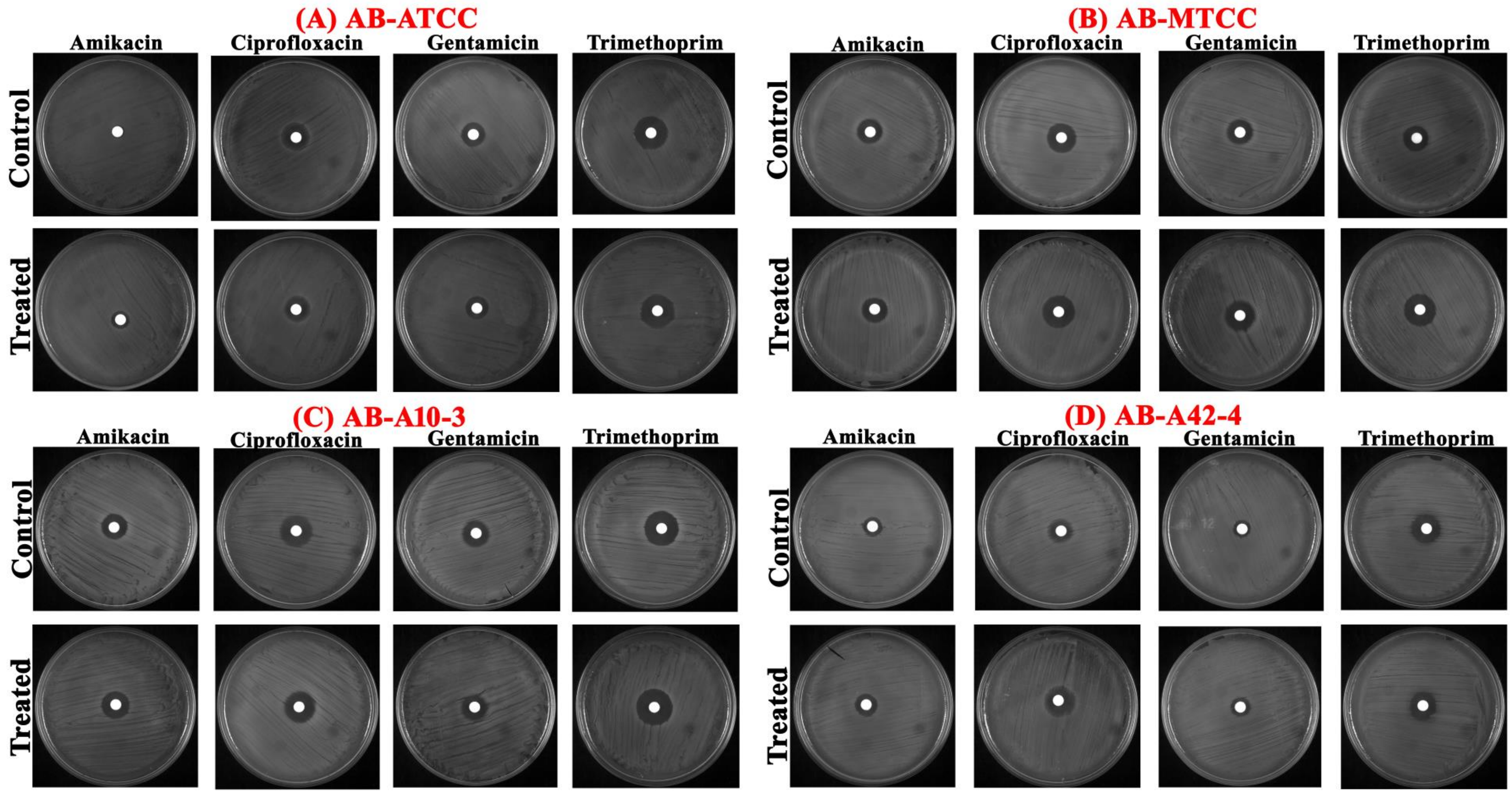

Supplementary Fig. 1: Effect of myrtenol at MBIC (200 µg/mL) on the susceptibility of AB strains to antibiotics. Disc diffusion plates showing the increased susceptibility of AB strains to antibiotics in the presence of myrtenol.
